# Annual acknowledgement of reviewers

**DOI:** 10.1186/s12862-016-0618-z

**Published:** 2016-02-29

**Authors:** Christopher Foote

**Affiliations:** BioMed Central, Floor 6, 236 Gray′s Inn Road, London, WC1X 8HB UK

## Abstract

The editors of *BMC Evolutionary Biology* would like to thank all our reviewers who have contributed to the journal in Volume 15 (2015).

**Matthew Aardema**

USA

**Amir Abbasi**

Pakistan

**Patrick Abbot**

USA

**Jessica Abbott**

Sweden

**Virginia Abdala**

Argentina

**Akio Abe**

Japan

**Thomas Abeli**

Italy

**M. Cristina Acosta**

Argentina

**Claudia Acquisti**

Germany

**Ingi Agnarsson**

USA

**Yasir Ahmed-Braimah**

USA

**Eyal Akiva**

USA

**Outi Ala-Honkola**

Finland

**Marisa Alarcón**

Spain

**Frank Albert**

USA

**Lorenzo Alibardi**

Italy

**Samuel Alizon**

France

**David Althof**

USA

**David Alvarez-Ponce**

USA

**Barbara Ambrose**

USA

**William Amos**

UK

**Valentin Amrhein**

Switzerland

**Katherine Amrine**

USA

**Erik Andersen**

USA

**Oivind Andersen**

Norway

**Erik Andersen**

USA

**Todd Anderson**

USA

**Jim Anderson**

Canada

**Carmelo Andujar**

Spain

**Maria Anisimova**

Switzerland

**Marta Antoniazzi**

Brazil

**Kevin Arbuckle**

UK

**Devin Arbuthnott**

Canada

**Miguel Arenas**

Spain

**Jeffrey Arendt**

USA

**Katherine Aronstein**

USA

**Nils Arrigo**

Switzerland

**Jonathan Atwell**

USA

**Frédéric Austerlitz**

France

**Hughes Austin**

USA

**Abdu Azad**

USA

**Ricardo Azevedo**

USA

**Takayuki Azuma**

Japan

**Wieslaw Babik**

Poland

**Lutz Bachmann**

Norway

**Wei-Ning Bai**

China

**Susan Bailey**

Denmark

**Scott Baird**

USA

**Amy Balanoff**

USA

**Guillaume Balavoine**

France

**Miguel Baltazar-Soares**

Germany

**Anna Bannikova**

Russian federation

**Sebastien Baratte**

France

**Maria Dolores Bargues**

Spain

**Annette Baudisch**

Germany

**Peter Baverstock**

Australia

**Arturo Becerra**

Mexico

**Timothy Beissinger**

USA

**Chuck Bell**

USA

**Kayce Bell**

USA

**Mark Belmonte**

Canada

**Jordan Bemmels**

USA

**Yoselin Benitez-Alfonso**

UK

**Elia Benito-Gutierrez**

Germany

**Mary Berbee**

Canada

**May Berenbaum**

USA

**Camillo Berenos**

UK

**Stewart Berlocher**

USA

**Giorgio Bertorelle**

Italy

**Alex Best**

UK

**Elvire Bestion**

UK

**Jong Bhak**

South Korea

**Leticia Bidegaray-Batista**

Spain

**Joseph Bielawski**

Canada

**Trine Bilde**

Denmark

**James Birchler**

USA

**Irene Bisang**

Sweden

**Christopher Blair**

USA

**Simon Blomberg**

Australia

**Astrid Boehne**

Switzerland

**Kirsten Bohn**

USA

**Alessandro Boianelli**

Italy

**Roberto Bonasio**

USA

**Ron Bonett**

USA

**Sarah Bordenstein**

USA

**Kevin Bown**

UK

**Michael Boyle**

Panama

**Robert Bradley**

USA

**Jason Bragg**

Australia

**Martin Brändle**

Germany

**Oskar Brattstrom**

UK

**Hans Breeuwer**

Netherlands

**Amanda Bretman**

UK

**Derek Briggs**

USA

**Carlos Briones**

Spain

**Dustin Brisson**

USA

**Samuel Brockington**

UK

**Lela Buckingham**

USA

**Ann F. Budd**

USA

**Emily Burdfield-Steel**

Finland

**Thorsten Burmester**

Germany

**Reto Burri**

Sweden

**Aodhan Butler**

Sweden

**Geraldine Butler**

Ireland

**Nick Butterfield**

UK

**Armando Caballero**

Spain

**Juliano Cabral**

Germany

**Patrick Calie**

USA

**Arley Camargo**

USA

**Leonardo Campagna**

Argentina

**Kevin Campbell**

Canada

**Lesley Campbell**

Canada

**Mauricio Canals**

Chile

**Simona Candiani**

Italy

**David Cannatella**

USA

**Chuan Cao**

UK

**Gamaliel Castañeda**

Mexico

**Jose Castresana**

Spain

**Dayaraj Cecilia**

India

**Robert Cerny**

Czech Republic

**Juliana Chacon**

USA

**Frederic Chain**

Canada

**Debapriyo Chakraborty**

USA

**Hue Sun Chan**

UK

**David Chapple**

Australia

**Mark Chase**

UK

**Raquel Chaves**

Portugal

**Jaime Chaves**

USA

**You-Hua Chen**

Canada

**Chen Chen**

China

**Shilin Chen**

China

**Tzen-Yuh Chiang**

Taiwan

**Les Christidis**

Australia

**Henry Chung**

USA

**Yujin Chung**

USA

**Alberto Civetta**

Canada

**Marcus Clauss**

Switzerland

**Lyndon Coghill**

USA

**Martin Cohn**

USA

**Jeff Cole**

UK

**David Collar**

USA

**Catherine Collins**

New Zealand

**Fabien Condamine**

Canada

**Piero Cossu**

Italy

**Christian Cox**

USA

**Cymon John Cox**

Portugal

**Keith Crandall**

USA

**Penelope Crane**

USA

**Kelly Craven**

USA

**Kate Crosby**

USA

**Eric Crubézy**

France

**Cameron Currie**

USA

**Asher Cutter**

Canada

**Thomas Cuypers**

Netherlands

**Tamás Czárán**

Hungary

**Luisa Dalla Valle**

Italy

**Giovanni Dall'Olio**

UK

**Marymegan Daly**

USA

**Anne C. Dalziel**

Canada

**Jakob Damgaard**

Denmark

**Etienne G.J. Danchin**

France

**Josephine Daub**

Spain

**Kalina Davies**

UK

**Shireen Davies**

UK

**Peter de Jong**

Netherlands

**Jill de Jong**

USA

**A. P. Jason de Koning**

Canada

**Ruud De Maagd**

Netherlands

**Thierry de Meeûs**

Burkina Faso

**Edivaldo de Oliveira**

Brazil

**L. de Oliveira**

Brazil

**Damien de Vienne**

France

**Jurriaan de Vos**

USA

**Bethany Dearlove**

UK

**Florence Debarre**

France

**Melanie Debiais-Thibaud**

France

**Jacquelin Defaveri**

Finland

**James Degnan**

Mexico

**Matthias Dehling**

Germany

**Jesse R.J. Delia**

USA

**Fargette Denis**

France

**Thomas Denk**

Sweden

**Dee Denver**

USA

**Jeremy Dettman**

Canada

**Kyle Dexter**

UK

**Raul Eduardo Diaz**

USA

**Antonio Diaz-Perez**

Venezuela

**Carmela Doenz**

Switzerland

**Martin Dohrmann**

USA

**Alex Dornburg**

USA

**Tim Downing**

Ireland

**Jeremy Draghi**

Canada

**Abby Drake**

USA

**Carlos Driscoll**

USA

**Guy Drouin**

Canada

**Zhiqiang Du**

USA

**Denis Duboule**

Switzerland

**Pablo Duchen**

Switzerland

**Sebastian Duchêne**

Australia

**Beth Dumont**

USA

**Robin Dunbar**

UK

**Elizabeth Duncan**

New Zealand

**David Dunigan**

USA

**Jason Dunlop**

Germany

**Katherine Dunn**

Canada

**Anne Duplouy**

Finland

**Alexander Duthie**

UK

**Muir Eaton**

USA

**Vinitha Ebenezer**

India

**Ingo Ebersberger**

Germany

**Scott Edwards**

USA

**Eran Elhaik**

UK

**Margaret Ellis**

USA

**Deena Emera**

USA

**Brent Emerson**

Spain

**Ove Eriksson**

Sweden

**Luis E. Escobar**

USA

**Hector Escriva**

France

**Marianne Espeland**

USA

**Rampal Etienne**

Netherlands

**Alistair Evans**

Australia

**Marie Fablet**

France

**Pierre-Henri Fabre**

France

**Jakob Fahr**

Germany

**Pengfei Fan**

China

**Mario Fares**

Ireland

**Zen Faulkes**

USA

**Marie-Julie Favé**

Canada

**Mario Fernández-Mazuecos**

UK

**Danilo Fernando**

USA

**M.M Ferrer**

Mexico

**Roberto Feuda**

USA

**Konrad Fiedler**

Austria

**Borja Figueirido**

Spain

**Jonathan Filee**

France

**Graziano Fiorito**

Italy

**Benjamin Fitzpatrick**

USA

**John Fitzpatrick**

UK

**Dina Fonseca**

USA

**Diego Fontaneto**

UK

**Vincent Foray**

Belgium

**Kristoffer Forslund**

Germany

**Jerome Foster**

USA

**Stephen Foster**

USA

**Christelle Fraisse**

UK

**Alain Franc**

France

**Loreta Freitas**

Brazil

**John V. Freudenstein**

USA

**Urban Friberg**

Sweden

**Claudia Fricke**

Germany

**Eyal Fridman**

Israel

**Matt Friedman**

UK

**Ville-Petri Friman**

UK

**M. L. Fritz**

USA

**Jinzhong Fu**

Canada

**Hironobu Fukami**

Japan

**Lisa Funkhouser-Jones**

USA

**Juergen Gadau**

USA

**Jennifer A. Gaddy**

USA

**Matthew Galaska**

USA

**Miguel Gallach**

Austria

**Brigitte Galliot**

Switzerland

**Nicolas Galtier**

France

**Guila Ganem**

France

**Hugo Gante**

Switzerland

**Ingrid Garbus**

Argentina

**Graciela García**

Uruguay

**Marc Garcia-Garcera**

France

**Alfred L. Gardner**

USA

**Michael Garratt**

Australia

**Philippe Gayral**

France

**Kerry Geiler-Samerotte**

USA

**David Gernandt**

Mexico

**Amanda Gibson**

USA

**Gary Gibson**

Canada

**Cara Gibson**

USA

**Phillip Gienapp**

Netherlands

**Danna Gifford**

UK

**Robert Gifford**

UK

**William Gilliland**

USA

**Janice Glime**

USA

**Jostein Gohli**

Norway

**Pablo Goicoechea**

Spain

**Zachariah Gompert**

USA

**Mary Gonder**

USA

**Fernando Gonzalez-Candelas**

Spain

**Toni Gossmann**

UK

**Marc Gottschling**

Germany

**Félix Goyache**

Spain

**Matthew Graham**

USA

**Catherine Graham**

USA

**Per Einar Granum**

Norway

**Dorde Grbic**

Switzerland

**Brian Greenhouse**

USA

**Anna Gregorio**

USA

**Gerald Grellet-Tinner**

USA

**C. S. Griffiths**

USA

**Astrid Groot**

Netherlands

**Thilo Gross**

UK

**Corinne Grover**

USA

**Catherine Grueber**

Australia

**Jared Grummer**

USA

**Raúl Guantes**

Spain

**Alain Guénoche**

France

**Eric Guilbert**

France

**Stephane Guindon**

New Zealand

**Ryan Gutenkunst**

USA

**V. A. Gvozdev**

USA

**Vaclav Gvozdik**

Czech Republic

**Wilfried Haetry**

UK

**Frank Hailer**

USA

**Nathaniel Hallinan**

USA

**Kurt Hammerschmidt**

Germany

**Vladimir Hampl**

Czech Republic

**Mira Han**

USA

**Charles Nathan Hancock**

USA

**Shinji Handa**

Japan

**James Hanken**

USA

**Les N. Harris**

Canada

**Phillip Harris**

USA

**Ellie Harrison**

UK

**Nils Hartmann**

Germany

**Simon Harvey**

UK

**Gerhard Haszprunar**

Germany

**Joachim Haug**

Germany

**Scott Hawley**

USA

**Pilar Haye**

Chile

**Alexander Hayward**

Sweden

**Einat Hazkani-Covo**

Israel

**Qixin He**

USA

**Denis Headon**

UK

**David Hearn**

USA

**Gerald Heckel**

Switzerland

**Abraham Hefetz**

Israel

**Andreas Hejnol**

Norway

**Ines Hellmann**

Germany

**Conrad Helm**

Germany

**Martin Helmkampf**

USA

**David Hembry**

USA

**Diana Hendrickx**

Netherlands

**Lars Hennig**

Switzerland

**John Heraty**

USA

**Sylvia Heredia**

Canada

**Ruth Hershberg**

Israel

**Attila Hettyey**

Hungary

**Andreas Heyland**

Canada

**Ana Hilario**

Portugal

**Khidir Hilu**

USA

**Axel Hochkirch**

Germany

**Sebastian Hoehna**

USA

**Anja Hoerger**

Austria

**Federico Guillermo Hoffmann**

USA

**Matthias Hoffmann**

Germany

**Mandë Holford**

USA

**Luke Holman**

Australia

**Rodney Honeycutt**

USA

**Steven Hoofer**

USA

**Andrew Hope**

USA

**Masaki Hoso**

Netherlands

**Tingjun Hou**

China

**Chu Hou**

Hong Kong

**Aaron Howard**

USA

**Y.K Hu**

China

**Xia Hua**

China

**Di-Ying Huang**

China

**Wen Huang**

USA

**Christian Huber**

USA

**Alan Hudson**

Sweden

**Jaime Huerta-Cepas**

Germany

**Larry Hufford**

USA

**Lily Hughes**

USA

**Martin Husemann**

Germany

**Sanna Huttunen**

Finland

**Ann Kathrin Huylmans**

Germany

**Atila Iamarino**

Brazil

**Hajime Ikeda**

Japan

**Juan Carlos Illera**

Spain

**Manuel Irimia**

Spain

**David Irwin**

Canada

**Marta Isasa**

USA

**Md Islam**

USA

**Andrzej Jagodzinski**

Poland

**Samuel James**

USA

**Timothy James**

USA

**Jukka Jernvall**

Finland

**Huifeng Jiang**

China

**Yu Jiang**

China

**Chris Jiggins**

UK

**Xiaohua Jin**

China

**Frida Johannesdottir**

USA

**Loretta Johnson**

USA

**Paul Johnston**

Germany

**Simon Joly**

Canada

**Ulf Jondelius**

Sweden

**Christopher Jones**

Canada

**Mathew Jones**

USA

**Jonathan Jones**

UK

**Lukas Kalous**

Czech Republic

**T.H Kao**

USA

**Jordan Karubian**

USA

**Brett A. Kaufman**

USA

**Jim Kaufman**

UK

**Joshka Kaufmann**

Germany

**Atsushi Kawakita**

Japan

**Alexander Keller**

Germany

**Clint Kelly**

Canada

**Nathan Kenny**

China

**Konstantin Khalturin**

Japan

**Aruna Kilaru**

USA

**Christopher Kimber**

Sweden

**Hirohisa Kishino**

Japan

**Ian Kitching**

UK

**Chris Knight**

UK

**Kevin Kocot**

USA

**Katia Koelle**

USA

**Bryan Kolaczkowski**

USA

**Miraim Konkel**

USA

**Irv Kornfield**

USA

**Etienne Kornobis**

Spain

**Helena Korpelainen**

Finland

**Jennifer Kovacs**

USA

**Indrikis Krams**

Estonia

**Henrik Krehenwinkel**

Germany

**Stacy Krueger-Hadfield**

USA

**Joachim Krug**

Germany

**Grzegorz Kudla**

USA

**Jonna Kulmuni**

UK

**Matjaž Kuntner**

Slovenia

**Shigehiro Kuraku**

Japan

**Joachim Kurtz**

Germany

**Ajjamada Kushalappa**

Canada

**Conrad Labandeira**

USA

**Benjamin Laenen**

Sweden

**Helene Lampe**

Norway

**Robert Lanfear**

Australia

**Richard Lankau**

USA

**Alice Lantinne**

Belgium

**Silvia Lanzavecchia**

USA

**Dan Larhammar**

Sweden

**Michael Lässig**

Germany

**Stephan Laurent**

Switzerland

**Sebastien Lavoue**

Taiwan

**Arnaud Le Rouzic**

France

**Jonathan Leake**

UK

**Chui Pin Leaw**

Malaysia

**R. Lecis**

Spain

**James Leebens-Mack**

USA

**David Legg**

UK

**Samuli Lehtonen**

Finland

**Jean-Francois Lemaitre**

France

**Tobias Lenz**

Germany

**Maryna Lesoway**

Canada

**Adrian Leuchtmann**

Switzerland

**Anthony Levasseur**

France

**Eli Levy**

Israel

**Paul Lewis**

USA

**Jessica Liberles**

USA

**Lisa Libungan**

Iceland

**Sun Liguang**

China

**Sergio Lima**

Brazil

**Willow Lindsay**

Sweden

**Susan Lingle**

Canada

**Cheng Liu**

China

**Irene Liu**

USA

**Kevin Liu**

USA

**Jian-Quan Liu**

China

**Laurence Loewe**

USA

**Josef Loidl**

Austria

**Philippe Lopez**

France

**Kay Lucek**

Switzerland

**Stefan Lupold**

Switzerland

**Mor Lurie-Weinberger**

Israel

**Samantha Lycett**

UK

**Philip Mabon**

Canada

**Regina Macedo**

Brazil

**Miloš Macholán**

Czech Republic

**Aide Macias-Muñoz**

USA

**D Luke Mahler**

USA

**Steven Manchester**

USA

**Judith Mank**

UK

**Paul Manos**

USA

**Michael Manuel**

France

**Kangshan Mao**

China

**Rajasekhar Maram**

USA

**Marina Marcet-Houben**

Spain

**Mark Margres**

Australia

**Karol Marhold**

Slovakia

**Frank Mari**

USA

**Luis Marins**

Brazil

**René Massimiliano Marsano**

Italy

**David Marshall**

UK

**Jeremy Marshall**

USA

**Sergio Martínez Cuesta**

UK

**Inigo Martinez-Solano**

Spain

**John P Masly**

USA

**Michael Matschiner**

Switzerland

**Brian Mautz**

Sweden

**Richard Mayden**

USA

**Stephen McCormick**

USA

**Stuart McDaniel**

USA

**Jenny McGuire**

USA

**Alesia McKeown**

USA

**Athena McKown**

Canada

**Ailsa McLean**

UK

**Paul McVeigh**

UK

**Thomas Mehner**

Germany

**Joana Meier**

Switzerland

**Amanda Melin**

USA

**José Melo-Ferreira**

Portugal

**Fernando Mendez**

USA

**John Mercer**

USA

**Vincent Merckx**

Netherlands

**Sami Merilaita**

Finland

**Margarita Metallinou**

USA

**Justin Meyer**

USA

**Michelle Meyer**

USA

**Neva Meyer**

USA

**Leithen M'Gonigle**

USA

**Johan Michaux**

Belgium

**Konstantin Mikhailov**

Russian federation

**Scott Miller**

USA

**M Mimura**

Japan

**Alessandro Minelli**

Italy

**Juan Marcos Mirande**

Argentina

**Dusan Misevic**

USA

**Charles Mitter**

USA

**Philipp Mitteroecker**

Austria

**Takahisa Miyatake**

Japan

**Amanda Moehring**

Canada

**Anders Pape Moller**

France

**Lionel Monod**

Switzerland

**Ludovica Montanucci**

Spain

**Michael Moody**

USA

**Paco Moore**

USA

**Yehu Moran**

Israel

**Alejandra Moreno-Letelier**

Mexico

**Claire Morgan**

UK

**Molly Morris**

USA

**Alan Moses**

Canada

**Christopher D. Moyes**

Canada

**Leonie Moyle**

USA

**Johannes Mueller**

Germany

**Sean Mullen**

USA

**Michael Mulligan**

USA

**Keith Murray**

USA

**Cesc Murria**

Spain

**Moritz Muschick**

UK

**Ville Mustonen**

UK

**Nicola Nadeau**

UK

**Indrajit Nanda**

Germany

**Alexander Nater**

Sweden

**David Nelson**

USA

**Zelong Nie**

China

**Claus Nielsen**

Denmark

**Nathan Nieto**

USA

**Naruo Nikoh**

Japan

**Harufumi Nishida**

Japan

**Ossi Nokelainen**

UK

**Jarle Nordeide**

Norway

**Reuben Nowell**

UK

**Robert Nudds**

UK

**Charles O’Kelly**

USA

**Christoph Oberprieler**

Germany

**Matthias Obst**

Sweden

**Matthew Ogburn**

USA

**Nancy Oguiura**

Brazil

**John Ohlrogge**

USA

**Paul Oliver**

Australia

**Samantha O'Loughlin**

UK

**Carrie Olson-Manning**

USA

**Chan Onn**

USA

**Juan Opazo**

Chile

**Alena Orlenko**

USA

**Juan Francisco Ornelas**

Mexico

**Pablo Orozco-Terwengel**

UK

**Javier Ortega Hernandez**

UK

**Elizabeth Ostrowski**

USA

**Donal O'Sullivan**

UK

**Oliver Otti**

Germany

**Chris Oufiero**

USA

**Israel Pagan**

Spain

**Luca Pagani**

Italy

**Louise Page**

Canada

**Anja Palandacic**

Austria

**Per Palsboll**

Netherlands

**Kristen Panfilio**

Germany

**Kevin Pang**

Norway

**Eric Pante**

France

**Mary-Lou Pardue**

USA

**Joong-Ki Park**

South Korea

**Steven Parratt**

Finland

**Luke Parry**

UK

**Marta Pascual**

Spain

**Rajeev Patnaik**

India

**Manus Patten**

USA

**Pavlos Pavlidis**

Greece

**Alexandra Pavlova**

Australia

**Jan Pawlowski**

Switzerland

**Andrea Paz**

Colombia

**Francisco Pedroche**

Mexico

**Kathryn Peiman**

Canada

**Nina Pekkala**

Finland

**Giuseppe Pellegrino**

Italy

**Leandro Pena**

Spain

**Diana Percy**

UK

**Costa Perdikaris**

Greece

**Susan Perkins**

USA

**Tim Perkins**

Australia

**Jean-Pierre Perrault**

Canada

**Mark Peterson**

USA

**Enrico Petretto**

USA

**Jobst Pfänder**

Germany

**Markus Pfenninger**

Germany

**Matt Phillips**

Australia

**Xavier Pico**

Spain

**Marcio Pie**

Brazil

**Simone Pigolotti**

Spain

**Andrea Pilastro**

Italy

**David Plachetzki**

USA

**Wojciech Plader**

Poland

**Alexander Platt**

USA

**Jörg Plötner**

Germany

**George Poinar**

USA

**John Pool**

USA

**Jaime Potti**

Spain

**Karen Pottin**

France

**Madison Powell**

USA

**Thomas Powell**

USA

**Luca Pozzi**

Germany

**Surendra K. Prajapati**

India

**Renae Pratt**

Australia

**Lorenzo Prendini**

USA

**Jill Preston**

USA

**Trevor Price**

USA

**Morgan Price**

USA

**Kathleen Prudic**

USA

**Oscar Puebla**

Germany

**Nalini Puniamoorthy**

USA

**Guenter Purschke**

Germany

**Andrey Puzachenko**

Russian federation

**R. Alexander Pyron**

USA

**Huan Qiu**

USA

**Andrea Quattrini**

USA

**Humberto Quesada**

Spain

**Jacek Radwan**

Poland

**Manon Ragonnet-Cronin**

UK

**Rajendhran Rajakumar**

USA

**Steven Ramm**

Germany

**Alex Rasnitsyn**

Russian federation

**Michael Raupach**

Germany

**David Reby**

UK

**Kathryn E. Reif**

USA

**Martin Reijns**

UK

**Tom Reimchen**

Canada

**James Davis Reimer**

Japan

**Jim Reist**

Canada

**Susanne Renner**

Germany

**Fabian Rentzsch**

Norway

**Dirk Repsilber**

Sweden

**Gustavo Requena**

Brazil

**Philipp Rescheneder**

Austria

**Ignacio Ribera**

Spain

**Gemma Richards**

Norway

**Chris Richards**

USA

**Jonathan Richardson**

USA

**Michael Richardson**

Netherlands

**Julia Richardson**

UK

**Stefan Richter**

Germany

**Dan Rigden**

UK

**David B. Rivers**

USA

**V.V. Robin**

India

**Estefania Rodriguez**

USA

**Silvia Teresa Rodriguez Ramilo**

Spain

**Francisco Rodriguez-Valera**

Spain

**Jeanne Romero-Severson**

USA

**Jonathan Romiguier**

France

**Vijendravarma Roshan**

Switzerland

**Eric Ross**

USA

**Louise Roth**

USA

**Sophie Rothammer**

Germany

**Carl Rothfels**

USA

**Alexandre Roulin**

Switzerland

**Jarko Routtu**

Germany

**Sean Rovito**

Mexico

**Melissah Rowe**

Norway

**Scott Roy**

USA

**Lukas Ruber**

Switzerland

**Manuel Ruedi**

Switzerland

**Michelina Ruocco**

Italy

**Anthony Russell**

Canada

**Joseph Ryan**

USA

**Carlos Saavedra**

Spain

**Ilik Saccheri**

UK

**Giuseppe Saccone**

Italy

**Ben Sadd**

USA

**Pablo Saenz**

Saudi Arabia

**Shota Sakaguchi**

Japan

**Keiko Sakakibara**

Japan

**Leonidas Salichos**

USA

**Daniele Salvi**

Portugal

**Pablo Sambucetti**

USA

**Diego San Mauro**

Spain

**Leonor Sanchez-Buso**

UK

**Thomas Sanger**

USA

**Eduardo Tarazona Santos**

USA

**Carlos Eduardo Sarmiento Monroy**

Colombia

**Michael Savageau**

USA

**Gerda Saxer**

USA

**Jeffrey Schank**

USA

**Helmut Schaschl**

Austria

**Alvin Schmaier**

USA

**Andres Schmitz**

Switzerland

**Gerald M. Schneeweiss**

Austria

**Kristan Schneider**

Austria

**Christine Schnitzler**

USA

**Sijmen Schoustra**

Netherlands

**Tanja Schwander**

Switzerland

**Erich Schwarz**

USA

**Klaus Schwenk**

Germany

**Jeremy Searle**

USA

**Elaine Seaver**

USA

**Jennifer Seddon**

Australia

**Kari Segraves**

USA

**Ryan Seipke**

UK

**Marie Semon**

France

**Andrea Sequeira**

USA

**Fernando Sequeira**

Portugal

**Eugene Shakhnovich**

USA

**Xingxing Shen**

USA

**Georgy Shenbrot**

Israel

**Guojun Sheng**

Japan

**Emma Sherratt**

Australia

**Tomohiko Shimada**

Japan

**Kentaro Shimizu**

Switzerland

**Alexander Shpakov**

Russian federation

**Susmita Shukla**

India

**Marcin Sielezniew**

Poland

**Paul Simion**

France

**James Simmer**

USA

**Ralph Simon**

Germany

**Andrew Simons**

USA

**Nadia Singh**

USA

**Jack Sites**

USA

**Claudio Slamovits**

Canada

**Paul Smaldino**

USA

**Emanuel Smeds**

Sweden

**Jouni Sorvari**

Finland

**Enrica Sozzi**

Italy

**Daniel Speiser**

USA

**Dieter Spiteller**

Germany

**Mark Springer**

USA

**Lewis Spurgin**

UK

**Gabor Sramko**

Hungary

**Michael Stat**

Australia

**Tristan Stayton**

USA

**Patrick Steinmetz**

Norway

**Per Stenberg**

Sweden

**Aleksandra Obrępalska-Stęplowska**

Poland

**Scott Steppan**

USA

**Eleanore Sternberg**

USA

**Jay Storz**

USA

**Johannes Strauss**

Germany

**Torsten Struck**

Norway

**Yoel Stuart**

USA

**Tomasz Suchan**

Poland

**Jan Suda**

Czech Republic

**Alfonso Susanna**

Spain

**Hitoshi Suzuki**

Japan

**Nicolas Svetec**

USA

**David L. Swanson**

USA

**Attila Szolnoki**

Hungary

**Denis Tagu**

France

**Mioko Taguchi**

Japan

**Koji Takayama**

Austria

**Nobuto Takeuchi**

Japan

**Ali Tarkan**

Turkey

**Bradford Taylor**

USA

**John Taylor**

Canada

**Michelle Taylor**

UK

**Javier Tello**

UK

**Olivier Tenaillon**

France

**Chunbo Teng**

China

**Jacob Tennessen**

USA

**Roger Thomas**

USA

**Paul Thomas**

USA

**Charles Thompson**

USA

**John Thompson**

USA

**Nelson Ting**

USA

**Ben Titus**

USA

**Christopher Toomajian**

USA

**Melissa Toups**

Switzerland

**Emmanuel Toussaint**

USA

**Simon Townsend**

Switzerland

**Ludwig Triest**

Belgium

**Jolyon Troscianko**

UK

**Devin Trudeau**

Israel

**Takashi Tsuchimatsu**

Japan

**Chie Tsutsumi**

Japan

**Tamir Tuller**

Israel

**Nobuko Tuno**

Japan

**Martin Turcotte**

Switzerland

**Seth Tyler**

USA

**Torbjorn Tyler**

Sweden

**Robert Unckless**

USA

**Nathan Upham**

USA

**Shunsuke Utsumi**

Japan

**Shinya Uwai**

Japan

**Filipa Vala**

Portugal

**Vera Lúcia Valente Gaiesky**

Brazil

**Joan Valles**

Spain

**Peter Vallo**

Czech Republic

**Ines Van Bocxlaer**

Belgium

**Arie van der Meijden**

Portugal

**Frans Van Roy**

Belgium

**Jean Vannier**

France

**Sergio Vargas**

Costa Rica

**Michael Veeman**

USA

**Patrick Venail**

Switzerland

**George Verboom**

South Africa

**Heleen Verlinden**

Belgium

**Cristina Vieira**

Portugal

**Lars Vilhelmsen**

Denmark

**Alfried Vogler**

UK

**Christoph von Beeren**

USA

**Michiel Vos**

UK

**Romé Voulhoux**

France

**Robert Vrijenhoek**

USA

**Hiroshi Wada**

Japan

**J. Wolfgang Wägele**

Germany

**Bruce Waldman**

New Zealand

**Patrick Walsh**

UK

**Cathy Walton**

UK

**Yongdong Wang**

China

**Hurng-Yi Wang**

Taiwan

**Harris Wang**

USA

**Yupeng Wang**

USA

**Wen Wang**

China

**Abigail Wark**

USA

**Rachel Warnock**

UK

**Ben Warren**

Switzerland

**Ian Warren**

USA

**Robert Waterhouse**

Switzerland

**Andrew Webb**

USA

**Claudia Weber**

USA

**Jessica Weber**

USA

**Christiane Weirauch**

USA

**Stephen G. Weller**

USA

**Jeff Wesner**

USA

**Lars Westberg**

Sweden

**Helena Westerdahl**

Sweden

**Christopher Wheat**

USA

**Geoff While**

Australia

**Michael Whitehead**

Australia

**Susan Whitehead**

USA

**Noah Whiteman**

USA

**Kenneth Whitney**

USA

**Camilla Whittington**

Australia

**Sébastien Wielgoss**

USA

**Jasmina Wiemann**

Germany

**Martin Wiemers**

Germany

**John Wiens**

USA

**Claus Wilke**

USA

**Jon Wilkins**

USA

**Endre Willassen**

Norway

**David Williams**

UK

**Tom Williams**

UK

**Anna Williford**

USA

**Damon Williford**

USA

**Joseph Wilson**

USA

**Mark Wilson**

Canada

**Chris Winchell**

USA

**Amanda Windsor**

USA

**Benjamin M. Winger**

USA

**Jamie Winternitz**

USA

**Jennifer Wisecaver**

USA

**Matthias Wolf**

Germany

**Yuri Wolf**

USA

**Joanna Wolfe**

USA

**Tim Wollesen**

Austria

**Katrine Worsaae**

Denmark

**Ada Wróblewska**

Poland

**Lin Xu**

USA

**Marna Yandeau-Nelson**

USA

**Ding Yang**

China

**Weizhao Yang**

Sweden

**Jian-Rong Yang**

USA

**Amir Yassin**

USA

**Laurel Yohe**

USA

**Takahiro Yonezawa**

China

**Atsuo Yoshido**

Czech Republic

**Douglas Yu**

UK

**Xue-Jie Yu**

USA

**Mika Zagrobelny**

Czech Republic

**Susanne Zajitschek**

Australia

**Christina Zakas**

USA

**Einat Zchori-Fein**

Israel

**Qing-Yin Zeng**

China

**Aibin Zhan**

China

**Wei Zhang**

USA

**Zhang Zhang**

China

**Dongyan Zhao**

USA

**Yan Zhong**

China

**Min Zhong**

USA

**Rongjia Zhou**

China

**Renchao Zhou**

China

**Marco Alberto Luca Zuffi**

Italy

